# The Vaccine Treatment of Gonococcal Arthritis

**Published:** 1908-04-18

**Authors:** 


### Points in Treatment.

### THE VACCINE TREATMENT OF GONOCOCCAL ARTHRITIS.

A good deal of work has already been done upon the treatment of gonorrhœa itself, and of other gonococcal affections, by means of vaccines. Dr. Cole and Dr. Meakins, for example, treated fifteen cases exclusively in this way, in order to test the efficacy of the vaccine unaided by other measures.

The gist of what has been found so far seems to be this: that the vaccine treatment requires no control by expensive opsonic index determinations; that it at least does no harm; that by itself it fails to cure in anything like a miraculous way; that nevertheless it is a real assistance in the cure, seeing that in some of the cases progress had been exceedingly slow previous to the use of the vaccine, whilst after its adoption recovery was greatly accelerated; and therefore that it is a procedure which is a valuable addition to other methods of dealing with the disease.

Vaccine treatment of all kinds is much more likely to come into use in general practice, when it is found that, for the particular kind of case it is proposed to treat, opsonic index determinations can be dispensed with.

It has been found that the best dose of vaccine to use to start with is that which contains the products of about 300 millions of dead gonococci; and the quantity of fluid required for the injection is something between five and fifteen drops, according to the concentration of the cultures. It is found that there is often a slight reaction at the site of injection some twelve to twenty-four hours after the latter has been given; but this reaction is comparatively unimportant, consisting as it does of a little redness and tenderness only, and these rapidly subside. No other ill-effects are observed.

The dose may be safely repeated in between seven and ten days' time, and the dosage may soon be doubled, so that the products of 600 millions of gonococci are being given each time. Some authors go on increasing the dose until it reaches the equivalent of 1,000 millions of the cocci; but as a usual thing a 600 million dose is sufficient.

It appears that the vaccines for gonococci, quite unlike those for staphylococci, do not require to be made from the patient's own organisms. The results are about the same whether the vaccine is prepared from the patient's gonococci or from a stock cultivation.

It is clear that if a patient is recovering well from gonococcal arthritis under ordinary measures no vaccine is necessary. It is equally clear that a lesion which originated as a gonococcal arthritic and has gone on to a stiff joint from periarticular fibrosis will not be much benefited by a vaccine—for the vaccines can only be expected to check the further inroads of gonococci themselves; they will not clear up fibrous tissue or make an ankylosed joint lissom again. It is when a case of undoubted gonococcal arthritis, proved to be so by its clinical relationship to a venereal discharge in which gonococci have been demonstrated, seems to be running an unfavourable course, that other forms of treatment are likely to be assisted by the use of a gonococcal vaccine; and in cases such as these it would probably be unwise to postpone the adoption of the vaccine treatment unduly.

